# Effects of Interval and Continuous Exercise Training on CD4 Lymphocyte Apoptotic and Autophagic Responses to Hypoxic Stress in Sedentary Men

**DOI:** 10.1371/journal.pone.0080248

**Published:** 2013-11-13

**Authors:** Tzu-Pin Weng, Shu-Chun Huang, Yu-Fen Chuang, Jong-Shyan Wang

**Affiliations:** 1 Healthy Aging Research Center, Graduate Institute of Rehabilitation Science, Chang Gung University, Tao-Yuan, Taiwan; 2 Department of Physical Medicine and Rehabilitation, Chang Gung Memorial Hospital, Tao Yuan, Taiwan; 3 Department of Rehabilitation Science, Jen-Teh Junior College of Medicine, Nursing and Management, Miaoli, Taiwan; Universidade de Sao Paulo, Brazil

## Abstract

Exercise is linked with the type/intensity-dependent adaptive immune responses, whereas hypoxic stress facilitates the programmed death of CD4 lymphocytes. This study investigated how high intensity-interval (HIT) and moderate intensity-continuous (MCT) exercise training influence hypoxia-induced apoptosis and autophagy of CD4 lymphocytes in sedentary men. Thirty healthy sedentary males were randomized to engage either HIT (3-minute intervals at 40% and 80%VO_2max_, n=10) or MCT (sustained 60%VO_2max,_ n=10) for 30 minutes/day, 5 days/week for 5 weeks, or to a control group that did not received exercise intervention (CTL, n=10). CD4 lymphocyte apoptotic and autophagic responses to hypoxic exercise (HE, 100W under 12%O_2_ for 30 minutes) were determined before and after various regimens. The results demonstrated that HIT exhibited higher enhancements of pulmonary ventilation, cardiac output, and VO_2_ at ventilatory threshold and peak performance than MCT did. Before the intervention, HE significantly down-regulated autophagy by decreased beclin-1, Atg-1, LC3-II, Atg-12, and LAMP-2 expressions and acridine orange staining, and simultaneously enhanced apoptosis by increased phospho-Bcl-2 and active caspase-9/-3 levels and phosphotidylserine exposure in CD4 lymphocytes. However, five weeks of HIT and MCT, but not CTL, reduced the extents of declined autophagy and potentiated apoptosis in CD4 lymphocytes caused by HE. Furthermore, both HIT and MCT regimens manifestly lowered plasma myeloperoxidase and interleukin-4 levels and elevated the ratio of interleukin-4 to interferon-γ at rest and following HE. Therefore, we conclude that HIT is superior to MCT for enhancing aerobic fitness. Moreover, either HIT or MCT effectively depresses apoptosis and promotes autophagy in CD4 lymphocytes and is accompanied by increased interleukin-4/interferon-γ ratio and decreased peroxide production during HE.

## Introduction

High-intensity exercise has been suggested to elicit greater aerobic and cardiovascular adaptations than low and moderate levels of exercise [1]. However, physical exercise imposes, paradoxically, both enhancing and impairing effects on immunity, depending on the type and intensity of exercise [2,3]. Lymphocyte-related immune dysfunction has been diagnosed in hypoxemic individuals suffering from cardiopulmonary disorders [4] or exposed to hypoxic environments such as high altitudes [5]. Blood is subjected to oxidative stress during extreme hypoxic exposure [6]. Excessive exposure to oxidative stress facilitates the programmed death of lymphocytes [7,8], resulting in increased risk of infectious diseases and autoimmune disorders. To the best of our knowledge, the exercise patterns that improves physical fitness and simultaneously increases the resistance to lymphocyte death provoked by hypoxia has not yet been established. In particular, the effect of high-intensity interval training (HIT) on immune regulation to date has not yet been studied.

Autophagy is important for regulating mitochondrial bioenergetics, proliferation, and programmed death of CD4 lymphocyte [9,10]. T help cell 1 (Th1) cytokine interferon-γ (IFN-γ) up-regulates autophagy of CD4 lymphocyte, whereas Th2 cytokine interleukin-4 (IL-4) counteracted IFN-γ–induced the cell autophagy [11,12]. Impaired autophagy leads to mitochondrial dysfunction and subsequently elevates oxidative stress, which may, in turn, trigger apoptotic cascade [13]. However, extreme autophagy causes cell death through excessive cellular digestion [14], and can also activate apoptosis [15]. Some evidences, including our previous findings, indicated that acute strenuous exercise down-regulated mitochondrial biogenesis, consequently activating programmed death pathways in lymphocytes [2,16]. However, exercise preconditioning may also protect tissue against ischemia-reperfusion injury by depressing leukocyte-derived oxidants [17,18]. 

Warm-up exercise (40%VO_2max_) has been observed to reduce vigorous exercise (80%VO_2max_)-induced risks of thrombosis associated with leukocyte and platelets under inflammatory conditions, this effect being a form of preconditioning [18]. Recently, clinical investigations revealed that high-intensity interval training (HIT) that consists of alternating low (40%VO_2max_)- and high (80%VO_2max_)-intensity exercise effectively suppressed oxidative stress/inflammation associated with hemodynamic/hemorheological dysfunctions [19,20]. Moreover, the authors’ study demonstrated that exercise training under hypoxic condition reduced senescent T-lymphocyte subsets with increasing IFN-γ level in blood, which responses were accompanied by depressed oxidative stress and pro-inflammatory cytokine production [21]. Accordingly, we further hypothesize that HIT influences hypoxia-induced apoptosis and autophagy of CD4 lymphocytes by modulating circulatory Th1/Th2 cytokines and redox status.

To answer the above questions, this investigation assessed how HIT (alternating 40% and 80%VO_2max_) and moderate intensity-continuous training (MCT, sustained 60%VO_2max_) for 5 weeks affected (i) corresponding biological markers of apoptosis and autophagy, (ii) plasma IFN-γ and IL-4 levels that reflected the regulation of the Th1/Th2 balance, and (iii) pro-inflammatory cytokine and peroxide productions during hypoxic (12%O_2_) exercise (HE). The goal of this study was to compare the effect of HIT and MCT on aerobic capacity improvement and their influence for modulating autophagy and apoptosis of CD4 lymphocyte.

## Methods

### Subjects

In total, there are thirty sedentary healthy men volunteered to participate in this study. All subjects were requested to be free of chronic pulmonary, cardiovascular, immune, metabolic, hemostatic and anemic diseases. Moreover, no subjects had engaged in any regular physical activity (i.e., exercise frequency ≦1 time/week, duration <20 min) for at least 1 year, or had taken dietary vitamins/ prophylactic anti-inflammatory medications 2 weeks before the study. All subjects were randomly divided into three groups: high-intensity interval training (HIT, n=10), moderate continuous training (MCT, n=10) and control (CTL, n=10) groups. The experiment protocols adopted for the study has been approved by Chang Gung Memorial Hospital Institutional Review Board Committee, and all subjects have completed signing the written informed consents after the experimental procedures were explained. This research design was performed in accordance with the ethical standards of the Declaration of Helsinki. Anthropometric and respiratory/cardiovascular characteristics of all individuals did not differ significantly before interventions (Table 1). Participants were instructed to fast for at least 8 hours and to refrain from strenuous physical exercise for at least 48 hours before sampling. All subjects arrived at the testing center at 8:30 AM to eliminate any possible diurnal effect.

**Table 1:** Basic characteristics of anthropometrics and cardiopulmonary fitness in various groups.

	**HIT**		**MCT**		**CTL**	
	**Pre**	**Post**	**Pre**	**Post**	**Pre**	**Post**
***Anthropometrics***						
**Age** (**yr**)	22.3±0.2		22.5±1.0		22.4±0.9	
**Height** (**cm**)	171.3±2.1		172.1±1.2		171.6±2.1	
**Weight** (**kg**)	67.5±2.7	65.7±2.3	66.1±1.8	65.8±2.1	64.8±2.1	65.5±2.1
**BMI** (**kg/m^2^**)	22.7±0.9	22.2±0.9	22.5±0.7	22.2±0.7	22.3±0.5	22.3±0.6
***Cardiopulmonaryfitness***						
***Ventilatory threshold***						
**Workload** (**watt**)	114±5	149±5^+‡^	113±6	140±5^+‡^	121±2	118±6
**Workload_peak_ (%**)	58.2±2.1%	62.1±2.3%^+^	58.9±2.5%	64.8±2.8%^+^	61.1±2.1%	59.9±2.1%
**V_E_** (**L/min**)	45.3±3.0	58.5±3.5^+#‡^	41.4±3.5	53.5±3.6^+‡^	45.1±2.5	44.7±2.3
**VO_2_** (**ml/min/kg**)	26.4±1.2	34.6±1.8^+#‡^	26.8±1.1	32.1±1.4^+‡^	28.2±1.1	27.8±1.2
**VO_2peak_** (**%**)	56.8±2.8%	59.8±2.1%^+^	57.9±2.2%	61.8±2.3%	61.4±2.2%	62.1±2.4%
**VCO_2_** (**ml/min/kg**)	27.5±1.4	34.7±1.8^+#‡^	28.4±1.4	32.3±1.6^+‡^	28.5±1.0	27.9±1.3
**HR** (**bpm**)	151±5	156±4	149±4	154±3	149±3	149±4
**SV** (**ml**)	83.4±3.8	93.3±3.7^+#‡^	84.5±2.4	88.9±3.6	86.7±3.4	86.3±4.2
**CO** (**L/min**)	12.4±0.4	14.6±0.6^+#‡^	12.3±0.4	13.6±0.5^+‡^	13.0±0.6	12.8±0.4
**MAP** (**mmHg**)	122±3	120±3	120±3	120±3	121±5	119±3
**SVC** (**mL/min/mmHg**)	102.7±3.0	120.4±4.2^+#‡^	103.1±2.9	113.3±4.1^+‡^	104.2±3.5	104.6±4.4
***Peak performance***						
**Workload** (**watt**)	196±6	240±7^+#‡^	192±4	216±7^+‡^	198±6	197±8
**V_E_** (**L/min**)	111.6±5.2	138.8±4.9^+#‡^	110.5±5.2	126.7±5.3^+‡^	115.2±6.4	117.0±6.2
**VO_2_** (**ml/min/kg**)	46.5±1.7	57.9±1.6^+#‡^	46.3±1.5	51.9±1.3^+‡^	45.9±1.7	44.4±1.8
**VCO_2_** (**ml/min/kg**)	56.6±2.2	67.8±2.1^+#‡^	55.0±1.7	62.7±1.6^+‡^	57.2±2.0	56.1±2.2
**HR** (**bpm**)	197±2	197±1	196±3	197±2	196±2	197±2
**SV** (**ml**)	76.2±4.5	93.5±2.1^+#‡^	78.8±3.2	85.3±5.1^+‡^	75.7±2.4	72.3±3.3
**CO** (**L/min**)	14.9±0.9	18.5.±0.8^+#‡^	15.1±0.6	16.8±0.7^+‡^	14.3±0.6	13.9±0.7
**MAP** (**mmHg**)	135±5	137±3	133±3	134±3	132±2	132±5
**SVC** (**mL/min/mmHg**)	110.3±4.5	135.0±4.2^+#‡^	113.5±4.9	123.2±6.7^+‡^	108.3±5.2	106.3±3.5

Values were mean±SE. HIT, high-intensity interval training group; MCT, moderate continuous training group; CTL, control group; Pre, pre-intervention; Post, post-intervention; BMI, body mass index; **V**_E_, minute ventilation; **V**O_2_, oxygen consumption; **V**CO_2_, carbon dioxide production; HR, heart rate; SV, stroke volume; CO, cardiac output; MAP, mean arterial pressure; SVC: systemic vascular conductance. n=10 in each group. ^+^P<0.05, Pre vs. Post; ^#^P<0.05, HIT vs. MCT. ‡P<0.05, HIT or MCT vs. CTL

### Training protocols

Both HIT and MCT groups performed training regimens on a stationary bicycle ergometer 5 times a week for 5 weeks. HIT subjects warmed up for 3-min at 30% of maximal O_2_ consumption (VO_2_max) before continue to five exercise cycles, each 3-min at 80% of VO_2max_ interspersed with a 3-min active recovery at 40% of VO_2max_. The exercise session was terminated by 3-minute cool-down at 30% of VO_2max_. The MCT group had the same warm-up and cool-down protocols as the HIT group except the training period were 30 minutes at 60% of VO_2max_ [19,20]. For comparison purpose, CTL participants did not any receive exercise but were carefully monitored and recorded the information of physical activity and healthy eating. Each subject used a heart rate (HR) monitor (Tango, SunTech Medical, UK) to obtain the assigned intensity of exercise. Borg 6-to-20 scale was used to assess the rate of perceived exertion during each 3 min testing and after each exercise session. The work-rate of bicycle ergometer was adjusted continuously to ensure that the intensity of exercise matched the target HR throughout the training period. The two exercise protocols were isovolumic at the same exercise duration [i.e. HIT exercise volume: 6 min (40% VO_2max_ + 80% VO_2max_) × 5 cycles) = MCT exercise volume: 30 min (60% VO_2max_)]. The CTL group kept original diets and physical activity habits for 5 weeks. All subjects recorded their daily activities and nutrition intakes with questionnaires throughout the experiment. These participants were instructed to refrain from extra regular exercise until the end of this study. Moreover, their compliance rates to the three interventions were 100%.

### Graded exercise tests

**Figure 1:**
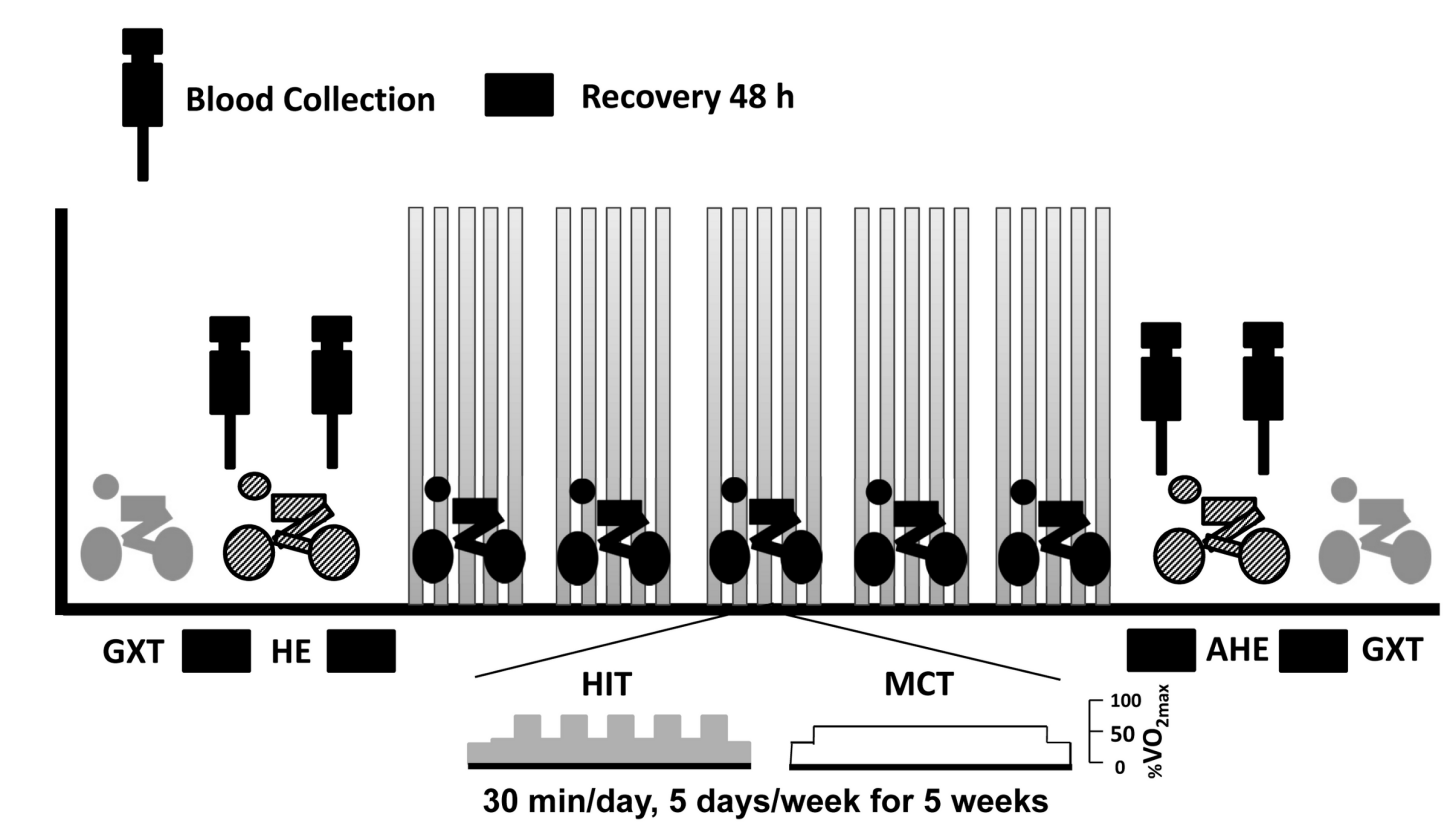
Design and time course of the experiment. Subjects (n=30) were randomly divided into three groups: control (CTL; n=10), high-intensity interval training (HIT, n=10), and moderate continuous training (MCT, n=10). Subjects in three groups were confront inactive (CTL group), or trained on a bicycle ergometer for five cycles 3-min at 80% of VO_2max_ interspersed with a 3-min active recovery at 40% of VO_2max_ (HIT group), or continuous at 60% of VO_2max_ for 30 min/day, 5 days/week, for 5 weeks. Each subject had to perform 1) graded exercise tests (GXT) 4 days before and 4 days after the intervention, 2) hypoxic exercise test (HE) on the second day before and on the second day after the intervention. At rest and immediately after the HE test, blood samples were collected. Grey cyclist, graded exercise test; striped cyclist, hypoxic (12%O_2_) exercise test; black cyclist, 5-week exercise intervention.

Each subject performed a graded exercise test (GXT) on a bicycle ergometer (Corival 400, Lode) to assess their aerobic fitness and hemodynamic functions 4 day before and 4 day after the 5-week interventions (Figure 1). The GXT comprised 2 minutes of unloaded pedaling followed by a continuous increase in work-rate of 20~30 W per 3 minute until exhaustion (i.e., VO_2max_). Minute ventilation (V_E_), VO_2_, and carbonic dioxide production (VCO_2_) were measured breath by breath using a computer-based system (PowerLab ML870, ADInstruments). Mean arterial pressure (MAP) was measured using an automatic blood pressure system (Tango, SunTech Medical, UK) and arterial O_2_ saturation was monitored by finger pulse-oximetry (model 9500, Nonin Onyx, Plymouth, Minnesota) [19,20]. The VO_2max_ was defined by the following criteria: (i) the level of VO_2_ increased less than 2 mL/kg/min over least 2 min; (ii) HR exceeded its predicted maximum; (iii) the respiratory exchange ratio exceeded 1.2, and (iv) the venous lactate concentration exceeded 8 mM, consistent with the guidelines of American College of Sports Medicine for exercise testing [22]. The value of ventilatory threshold was determined by two experienced, independent reviewers using the V-slope method by applying the following ventilatory criteria; (i) the V_E_/VO_2_ ratio increased without a corresponding increase in the V_E_/VCO_2_ ratio; (ii) PETO_2_ increased without a decrease in the PETCO_2_, or (iii) a departure from linearity for VE [22].

### Hypoxic exercise (HE) tests and blood collection

Each subject performed the HE test 2 days before the intervention. A subsequent 2^nd^ HE test was conducted 2 days after the intervention in an air-conditioned normobaric hypoxia chamber (Colorado Mountain Room, Boulder, CO), as mentioned in our previous studies (Figure 1) [21,23]. The hypoxia chamber was maintained at a temperature of 22 +/- 0.5°C with a relative humidity of 60+/- 5%; a CO_2_ scrubber eliminated CO_2_ in the air ( <3,500 ppm) [21,23]. The HE test on the bicycle ergometer required 50 W of warm-up for 3 min, an increase of work rate to 100 W of continuous exercise for 20 min, and then recovery to 50 W of cool-down for 3 min. During the test, the O_2_ concentration was set to 12%, which corresponds to altitudes of 4,460 m. 

At rest and immediately after the HE test, 40 mL of blood samples were collected from an antecubital vein using a clean venipuncture (20 gauge needle) under controlled venous stasis at 40 torr. The first 2 mL of blood was discarded, and the remaining blood was used to measure hematological parameters, CD4 lymphocyte functions, and plasma peroxide and cytokine levels. Blood cells were counted using a Sysmax SF-3000 cell counter (GMI, Inc., Ramsey, MN) [21,23].

### Cardiac hemodynamic measurements

All subjects were connected to the bioreactance-based cardiac output measurements device (NICOM, Cheetah Medical) that allowed continuous monitoring of cardiac hemodynamic functions throughout GXT, as described in our previous studies [19,20,24]. A radiofrequency generator for creating a high-frequency current is attached to the thorax through 4 dual-surface electrode stickers, and a receiving amplifier is included for recording the transthoracic voltage in response to the injected current. Briefly, the NICOM system analyzes time-dependent relative phase shifts (ΔФ) between the input signal relative to the output signal. Signals were applied to and recorded from the left and right sides of the thorax; these signals were processed separately and averaged after digital processing. Therefore, ΔФ is proportional to instantaneous changes of aortic blood flow [24]. Electrodes and cables were placed on the subject’s back to avoid disturbance for upper body motion. It has been shown that stroke volume (SV) was estimated using the following equation: SV=C×VET×dФ/dtmax, where C is a constant of proportionality, and VET denotes ventricular ejection time, which is obtained from the NICOM and ECG signals. The CO and systemic vascular conductance (SVC) were then calculated using the following equations: CO=SV×HR; SVC=CO/MAP.

### Lymphocyte isolation

 Blood samples (30 mL) were transferred to polypropylene tubes containing sodium citrate (3.8 g/dl: 1 vol. to 9 vol. of blood) (Sigma, St-Louis, MI). Peripheral blood lymphocytes were isolated by density-gradient centrifugation on lymphoprep tubes (Nycomed). Isolated cells were then washed three times in Roswell Park Memorial Institute (RPMI) medium (Gibco, Invitrogen; pH 7.4). The number of lymphocytes was adjusted using the RPMI medium to 2×10^6^ cells/ml [16,21,23]. All experiments processes in this study were conducted under a controlled environment to eliminate any uncertainty arising from human factor. The tests were repeated to ensure for reproducibility. 

### Autophagy-related markers

Lymphocytes (2×10^6^ cells/ml) were cultivated in culture medium [RPMI 1640 medium supplemented with 10% fetal bovine serum (Biological Industries), 1 mM glutamine (Sigma), 100 IU penicillin/streptomycin (Sigma)]. Subsequently, the cells were untreated (basal) or treated in the presence (Rap) or absence (Vehicle) of rapamycin (500nM, Cell Signaling) for 24 h at atmosphere of 5% CO_2_ at 37°C. All treated or untreated cells were double stained with allophycocyanin (APC)-CD4 bound to surface marker and with fluorescein isothiocyanate (FITC) bound to the cellular autophagic markers. Detail experimental procedures were as follows. The lymphocyte suspensions (2×10^6^ cells/ml) were incubated with a saturating concentration (10 μg/ml) of monoclonal anti-human CD4 antibody conjugated with APC (eBioscience) for 30 min at 4°C in the dark. After surface staining, 1 ml of commercial fixation buffer (eBioscience) was added to each sample, and these were incubated at room temperature for 20 min in the dark. Following fixation, the cells were washed twice and permeabilized using a commercial permeabilization washing buffer (eBioscience), and then incubated at 4°C for 30 min in the dark with a saturation concentration (10 μg/mL) of monoclonal anti-human mTOR (Cell Signaling), Beclin-1 (Novus Biologicals), Phospho-Bcl-2 (Cell Signaling), or LAMP-2 (eBioscience) monoclonal antibody conjugated with FITC, or anti-IgG (eBioscience) control antibody conjugated with FITC. Moreover, the second antibody termed donkey anti-goat IgG (Jackson Immuno Research Laboratories) is conjugated with FITC for binding to the anti-human Atg-1 (Cruz Marker), similarly antibody termed goat anti-rabbit IgG (Jackson Immuno Research Laboratories) is conjugated with FITC for binding with the LC3-II (Cell Signaling) and Atg-12 (Cell Signaling) in dark for 30 min at 4°C. 

Additionally, we have conducted immunostaining to obtain CD4/CD8 ratio. Finally, the lymphocytes treated with the control antibody were utilized to correct for background fluorescence, and the fluorescence gained from 10,000 events representing the lymphocytes was calculated using a FACScan flow cytometer (Becton Dickinson, franklin Lakes, NJ) [21,23]. 

### Acridine orange (AO) staining for acidic vesicular organelles

The volume of the cellular acidic compartment was increased in autophagy [9]. Hence, staining by acridine orange for the acidified autophagosome was used as an autophagy marker [9]. After CD4 antibody staining, the cells (2×10^6^ cells/ml) were incubated with medium containing 1 μg/mL acridine orange (Fluka BioChemika) for 15 minutes. Subsequently, cells were washed with PBS and the acidic vesicular organelles were determined by flow cytometry. A minimum of 10,000 cells within the gated region were analyzed.

### Lymphocyte apoptosis

Samples of lymphocytes (2×10^6^ cells/ml) were exposed to 500uM rapamycin in culture medium for 24 h at 37°C. Lymphocyte apoptosis was analyzed based on active caspase-3, caspase-9, and phosphatidyl serine (PS) exposure, as described in our previous studies [16,23].

#### (a): Detection of active caspase-3 and caspase-9

Active caspase-3 and caspase-9 contents in lymphocytes were detected by flow cytometry using commercial fluorescein active caspase kits (Mountain View) [16,23].

#### (b): Exposure of phosphatidylserine (PS)

This assay is based on changes in the cell surface exposure of PS during the early stages of apoptosis and the selective affinity of annexin V for this phospholipid. Detection of lymphocyte PS exposure was accomplished by flow cytometry using commercial annexin V kit (Mountain View) [16,23].

### Plasma IFN-γ, IL-4, myeloperoxidase (MPO), and IL-6 levels

From all subjects, an additional 5-mL blood samples was obtained, placed in a cold centrifuge tube containing EDTA (final concentration, 4 mM) (Sigma Chemical Co., Louis, MO 63178), and immediately centrifuged at 3000g for 10 min at 4 °C. The plasma samples were stored at -80°C until assay. Plasma IFN-γ (DIF-50, R&D systems, Minneapolis, MN), IL-4 (HS400, R&D systems, Minneapolis, MN), MPO (E-80PX, Immunology Consultants Labaory, Newberg, OR), and IL-6 (BMS213HS, eBioscience, San Diego, CA) concentrations were quantified by commercially available ELISA kits [21].

### Statistical analysis

Data were expressed as means±SE. Data analysis was performed using the statistical software package StatVeiw VI (SAS institute). Experimental results were analyzed by 3 (groups) x 4 (time sample points) repeated-measures ANOVA and Bonferroni’s post hoc test were performed to compare the hematological parameters, CD4 lymphocyte autophagy and apoptosis, as well as, plasma peroxide and cytokine levels before and immediately after HE at the beginning of the study and 5 weeks after various interventions. Additionally, sample size and effect size (Cohen’s d) were performed using the statistical software GPower 3.1. The statistical power is determine on 0.95 and the criterion for significance was P < 0.05. The autophagy/apoptosis related biomarkers were highly reproducible from day to day, and single measure intraclass coefficient correlation were from 0.94 and 0.92 in Day 0 and Day 1 for the test-retest reliability in this study.

## Results

### Cardiopulmonary fitness

Anthropometric variables did not significantly differ among the three groups (Table 1). Both HIT and MCT for the 5 weeks of training increased the work-rate, V_E_, VO_2_, and VCO_2_ at the ventilation threshold and peak performance (Table 1, P<0.05). For hemodynamic response, either HIT or MCT regimen also significantly increased SV, CO, and SVC during GXT (Table 1, P<0.05). Moreover, the HIT group exhibited a greater improvement in cardiopulmonary fitness than the MCT group (Table 1, P<0.05). However, CTL for 5 weeks did not influence these ventilatory and hemodynamic responses to GXT (Table 1).

### Leukocyte and lymphocyte subset counts

Acute HE significantly increased leukocyte, total lymphocyte, and CD8 lymphocyte counts and caused a decrease in the ratio of CD4 to CD8 lymphocytes (Table 2, P<0.05). However, the counts of leukocyte and various lymphocyte subsets before or after the HE test remained unchanged following the 5-week intervention with HIT, MCT, or CLT (Table 2).

**Table 2:** Effects of various interventions on leukocyte and lymphocyte subset counts in blood.

	**HIT**		**MCT**		**CTL**		
	**Pre**	**Post**	**Pre**	**Post**	**Pre**	**Post**	
**Leukocyte (×10^3^ cells/μL)**							
**Rest**	5.78±0.35	5.19±0.29	5.32±0.34	5.59±0.34	5.61±0.39	5.45±0.42	
**HE**	8.36±0.53*	7.26±0.46*	8.2±0.59*	7.45±0.36*	7.94±0.52*	7.91±0.36*	
**Total lymphocyte (×10^3^ cells/μL)**							
**Rest**	2.23±0.19	2.28±0.22	2.04±0.14	1.95±0.17	2.08±0.17	1.99±0.12	
**HE**	3.42±0.28*	3.16±0.26*	3.48±0.29*	3.08±0.29*	3.21±0.21*	3.22±0.12*	
**CD4 lymphocyte (×10^3^ cells/μL**)							
**Rest**	0.81±0.14	0.87±0.15	0.82±0.07	0.84±0.09	0.81±0.12	080±0.13	
**HE**	0.87±0.11	0.95±0.12	0.85±0.28	0.93±0.09	0.83±0.10	0.81±0.12	
**CD8 lymphocyte (×10^3^ cells/μL**)							
**Rest**	0.85±0.24	0.86±0.13	0.82±0.06	0.84±0.07	0.82±0.11	0.80±0.09	
**HE**	1.26±0.15*	1.36±0.28*	1.24±0.13*	1.32±0.06*	1.21±0.10*	1.22±0.12*	
**CD4/CD8 lymphocyte (ratio)**							
**Rest**	1.06±0.19	1.01±0.08	1.03±0.15	1.00±0.05	1.01±0.17		1.00±0.13
**HE**	0.70±0.10*	0.73±0.06*	0.66±0.18*	0.70±0.06*	0.71±0.12*		0.70±0.10*

Values were mean±SE. HIT, high-intensity interval training group; MCT, moderate continuous training group; CTL, control group; Pre, pre-intervention; Post, post-intervention; Rest, resting; HE, hypoxic (12%O_2_) exercise test. n=10 in each group. *P<0.05, Rest vs. HE; ^+^P<0.05, Pre vs. Post.

### Autophagy and apoptosis in CD4 lymphocytes

**Figure 2:**
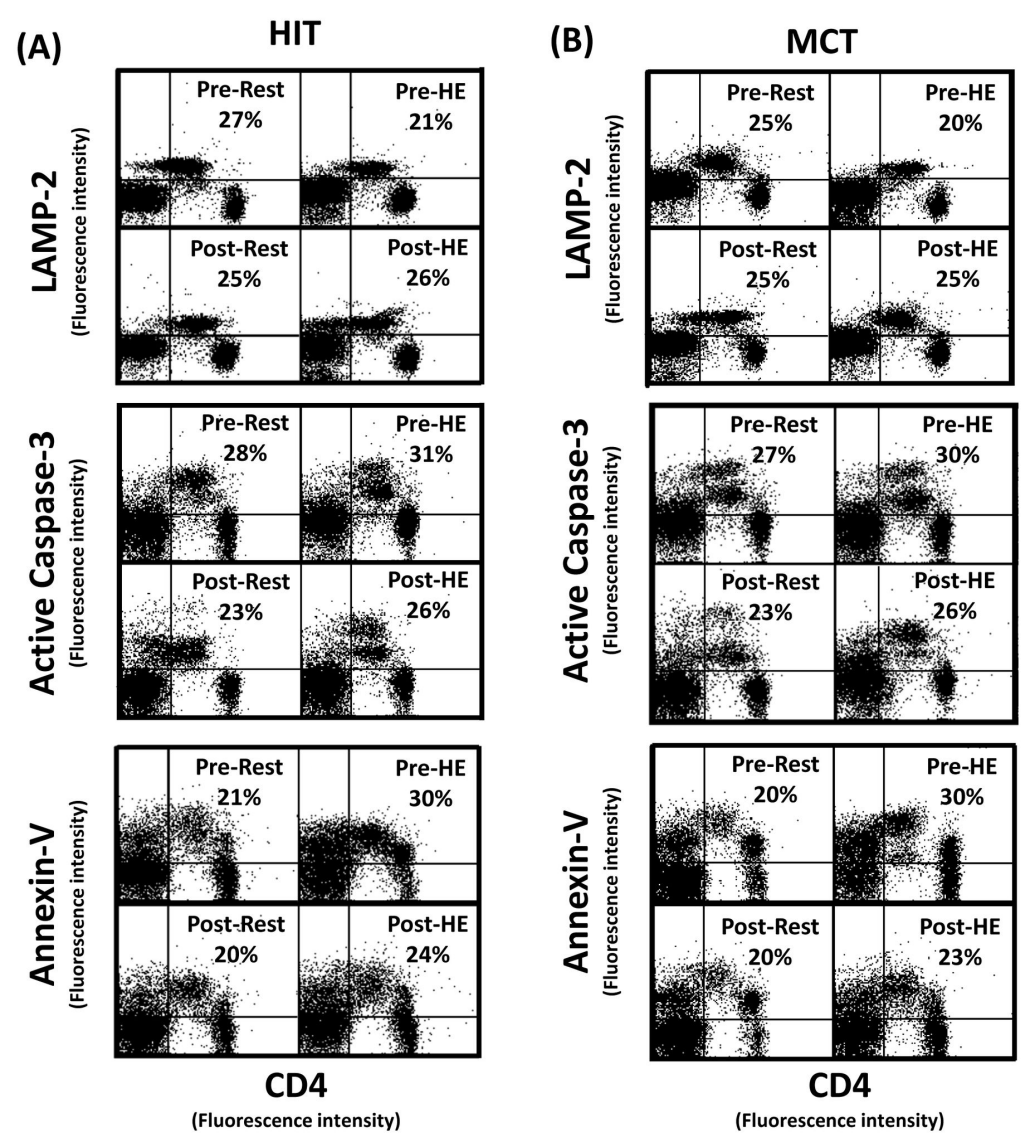
Flow cytometric analysis of apoptosis and autophagy in CD4 lymphocyte (Day 0). Graph showing the flow cytometric analysis of high intensity-interval (HIT) (**A**) and moderate intensity-continuous (MCT) (**B**) exercise training effects on main biomarkers of apoptosis and autophagy [i.e., LAMP-2, active caspase -3, and annexin V staining (phosphotidylserine exposure)] in untreated CD4 lymphocytes (Day 0). **Pre**, pre-intervention; **Post**, post-intervention; **Rest**, resting; **HE**, hypoxic (12%O_2_) exercise test**; Basal**, untreated CD4 lymphocytes (**Day 0**).

**Figure 3:**
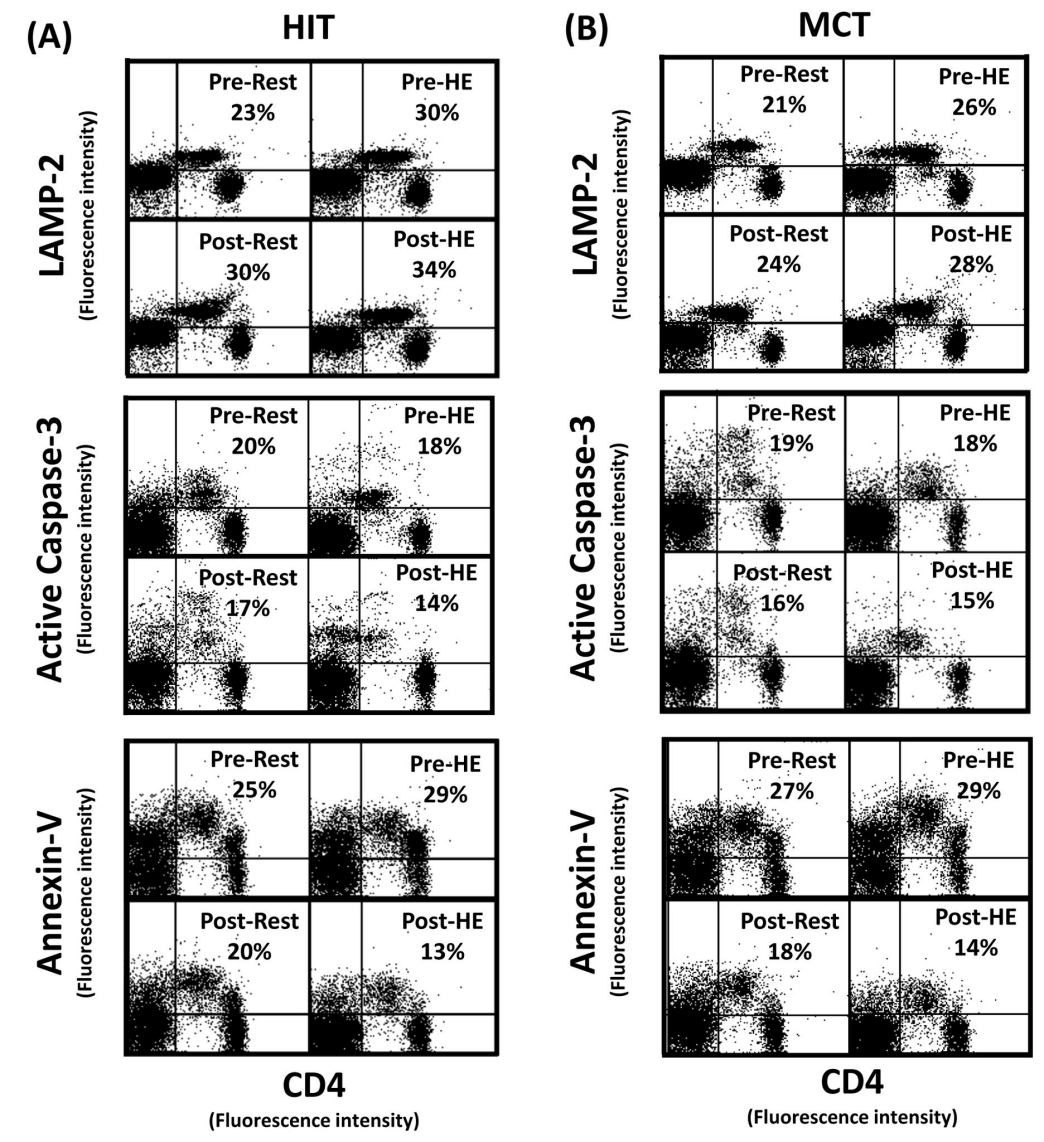
Flow cytometric analysis of apoptosis and autophagy in CD4 lymphocyte (Day 1). Graph showing the flow cytometric analysis of high intensity-interval (HIT) (**A**) and moderate intensity-continuous (MCT) (**B**) exercise training effects on main biomarkers of apoptosis and autophagy [i.e., LAMP-2, active caspase -3, and annexin V staining (phosphotidylserine exposure)] in rapamycin-treated CD4 lymphocytes (Day 1). **Pre**, pre-intervention; **Post**, post-intervention; **Rest**, resting; **HE**, hypoxic (12%O_2_) exercise test**; Basal**, untreated CD4 lymphocytes (**Day 0**).

**Figure 4:**
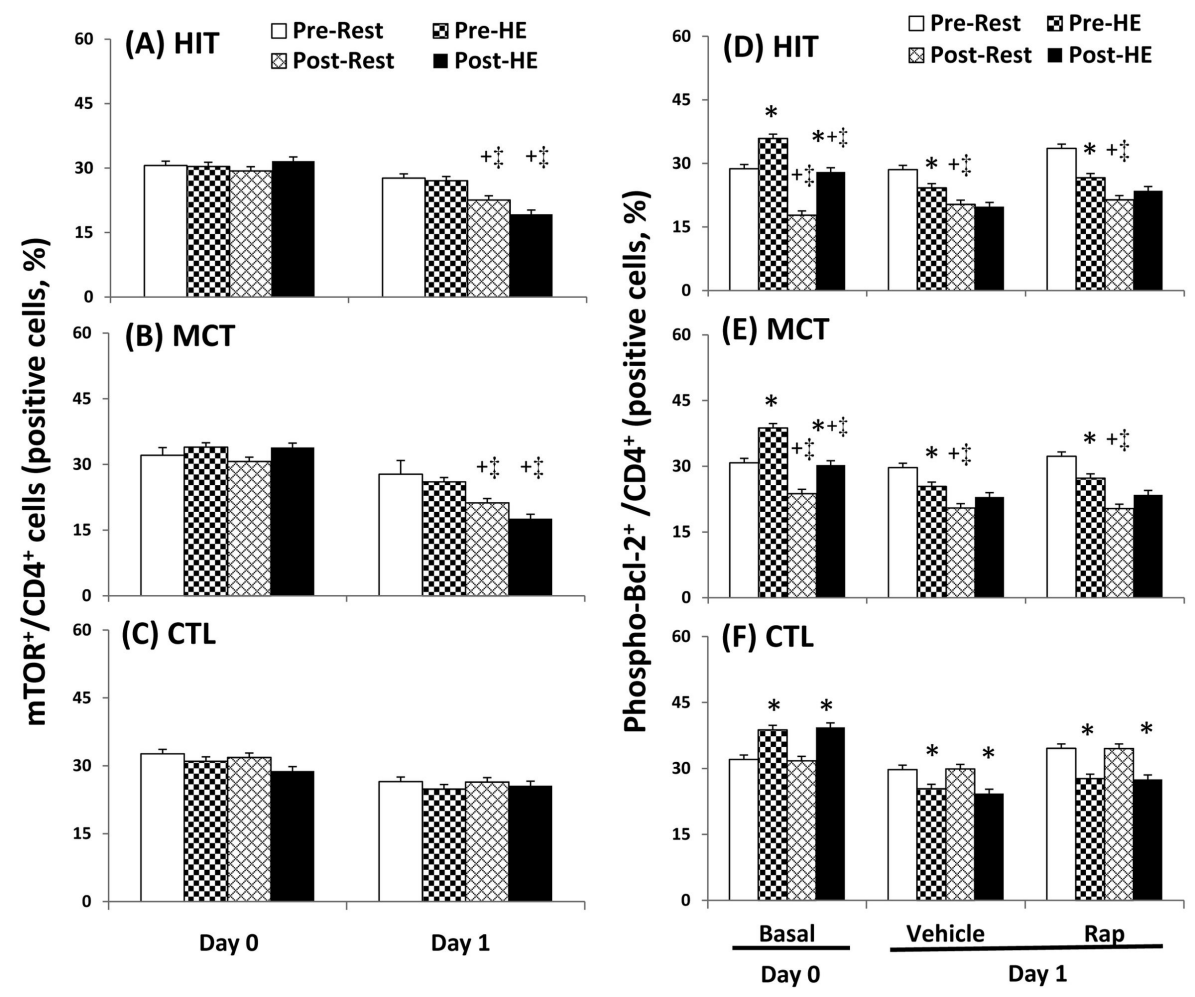
Effects of various interventions on levels of mTOR and phospho-Bcl-2 in CD4 lymphocytes. (**A**-**C**) mTOR; (**D**-**F**) phospho-Bcl-2; **HIT**, high-intensity interval training group (**A**, **D**); **MCT**, moderate continuous training group (**B**, **E**) ; **CTL**, control group (**C**, **F**)**; Pre**, pre-intervention; **Post**, post-intervention; **Rest**, resting; **HE**, hypoxic (12%O_2_) exercise test**; Basal**, untreated CD4 lymphocytes (**Day 0**); **Vehicle** and **Rap**, CD4 lymphocytes treated in the absence and presence of rapamycin (500nM) for 24 h (**Day 1**), respectively. *P<0.05, **Rest** vs. **HE**; +P<0.05, **Pre** vs. **Post**; ‡P<0.05, **HIT** or **MCT** vs. **CTL**. Values were mean±SE. n=10 in each group.

**Figure 5:**
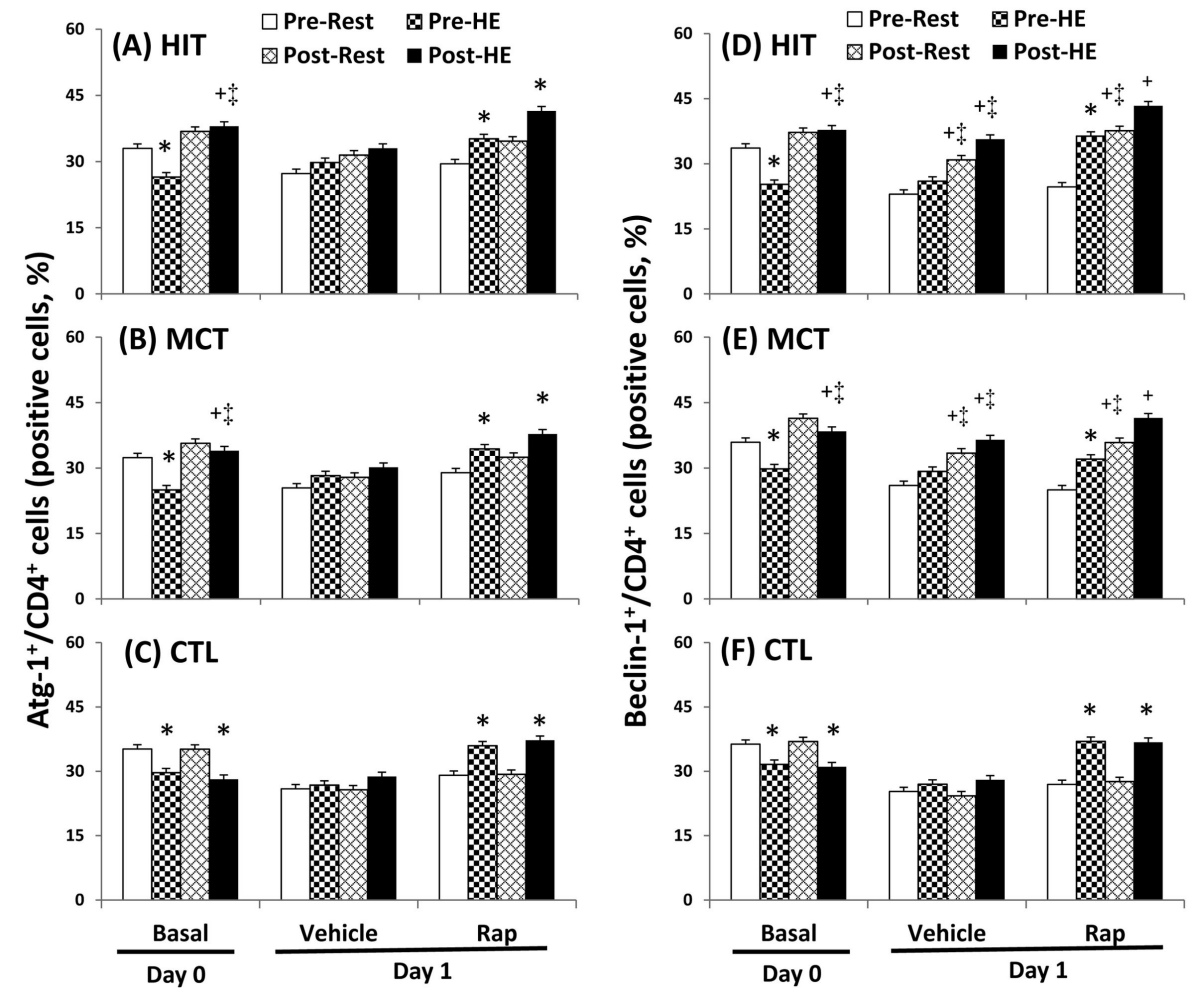
Effects of various interventions on levels of Atg-1 and Beclin-1 in CD4 lymphocytes. (**A**-**C**) Atg-1; (**D**-**F**) Beclin-1; **HIT**, high-intensity interval training group (**A**, **D**); **MCT**, moderate continuous training group (**B**, **E**) ; **CTL**, control group (**C**, **F**)**; Pre**, pre-intervention; **Post**, post-intervention; **Rest**, resting; **HE**, hypoxic (12%O_2_) exercise test**; Basal**, untreated CD4 lymphocytes (**Day 0**); **Vehicle** and **Rap**, CD4 lymphocytes treated in the absence and presence of rapamycin (500nM) for 24 h (**Day 1**), respectively. *P<0.05, **Rest** vs. **HE**; +P<0.05, **Pre** vs. **Post**; ‡P<0.05, **HIT** or **MCT** vs. **CTL**. Values were mean±SE. n=10 in each group.

**Figure 6:**
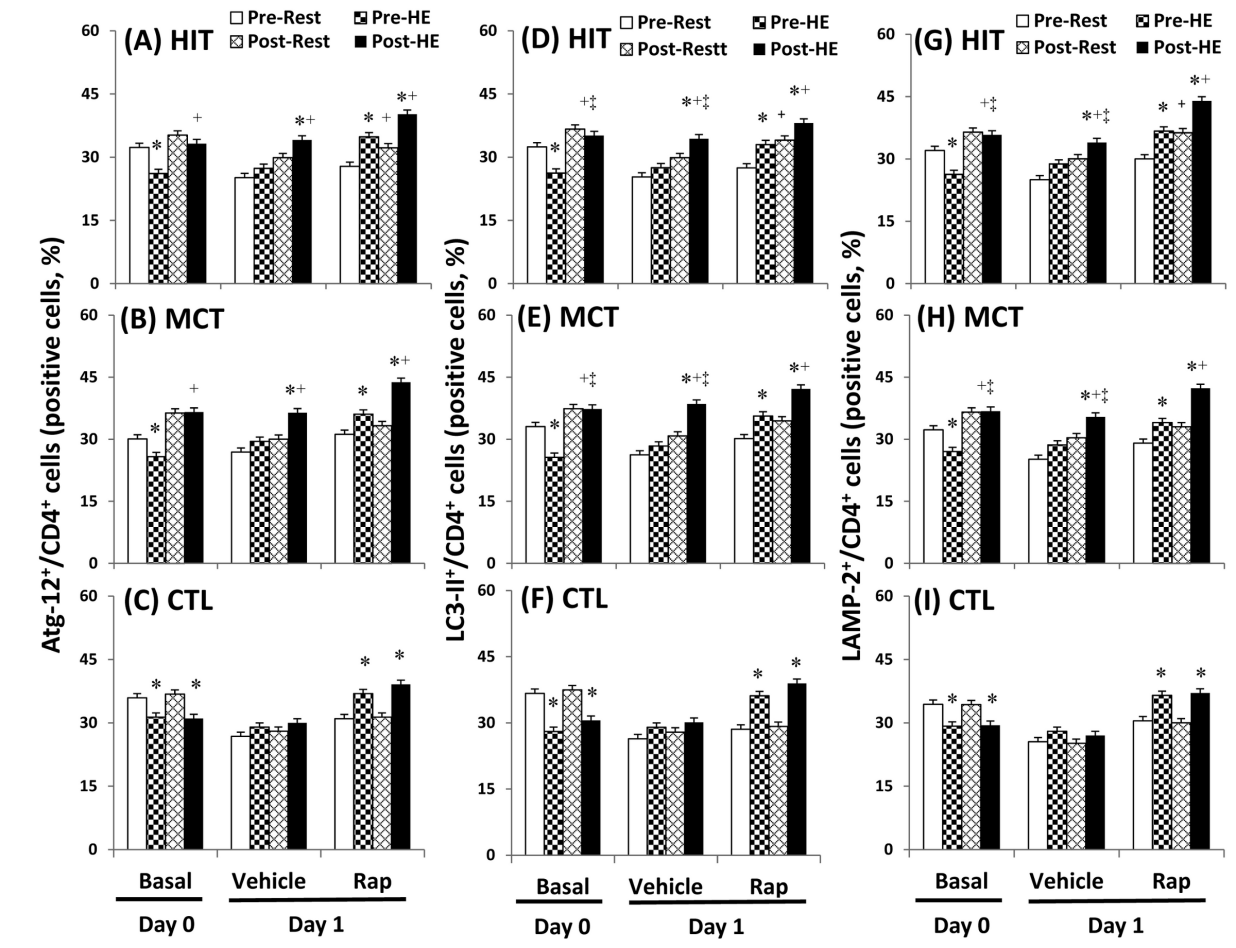
Effects of various interventions on levels of Atg-12, LC3-II, and LAMP-2 in CD4 lymphocytes. (**A**-**C**) Atg-12; (**D**-**F**) LC3-II; (**G**-**I**) LAMP-2; **HIT**, high-intensity interval training group (**A**, **D**, **G**); **MCT**, moderate continuous training group (**B**, **E**, **H**)**; CTL**, control group (**C**, **F**, **I**)**; Pre**, pre-intervention; **Post**, post-intervention; **Rest**, resting; **HE**, hypoxic (12%O_2_) exercise test**; Basal**, untreated CD4 lymphocytes (**Day 0**); **Vehicle** and **Rap**, CD4 lymphocytes treated in the absence and presence of rapamycin (500nM) for 24 h (**Day 1**), respectively. *P<0.05, **Rest** vs. **HE**; +P<0.05, **Pre** vs. **Post**; ‡P<0.05, **HIT** or **MCT** vs. **CTL**. Values were mean±SE. n=10 in each group.

**Figure 7:**
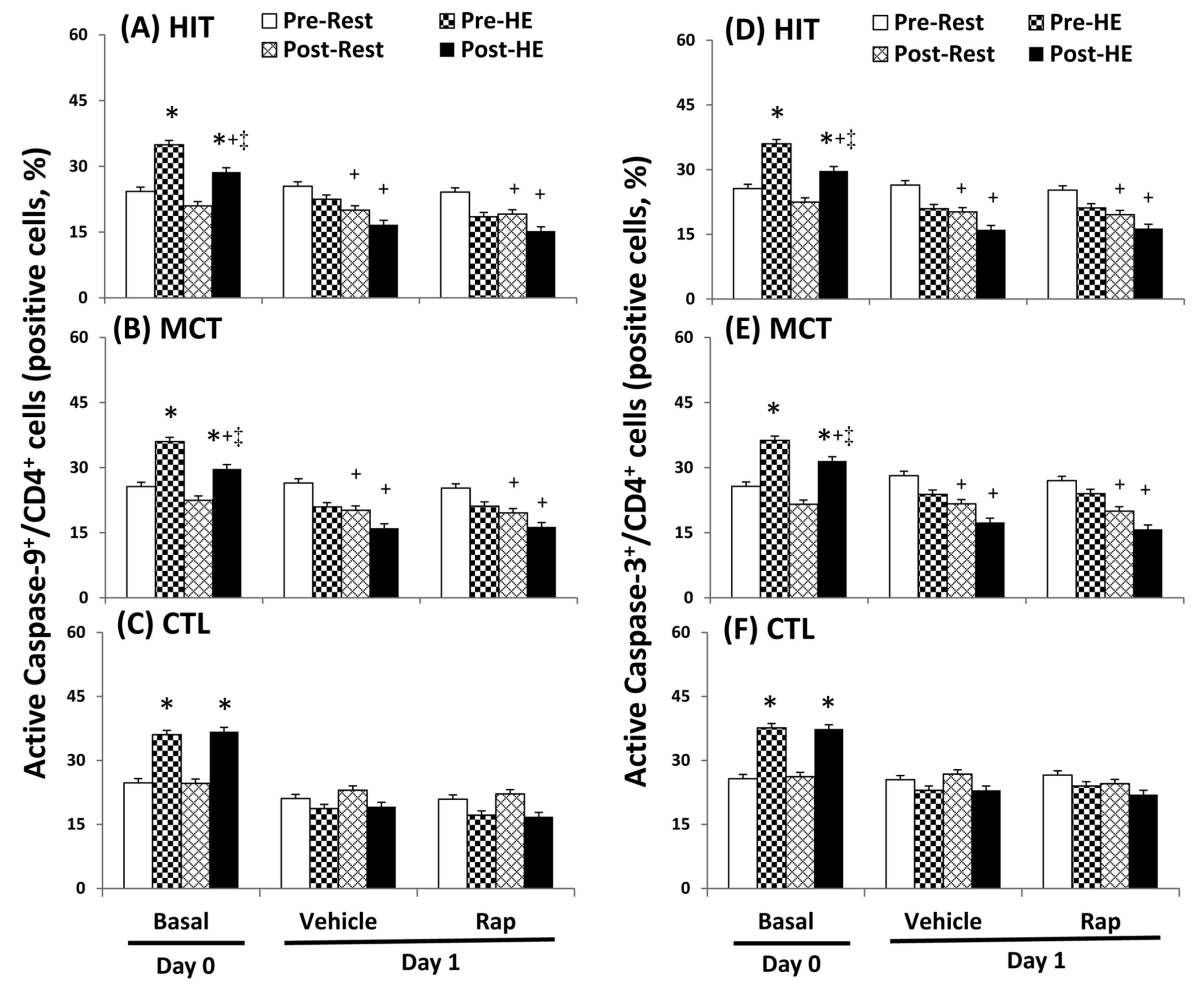
Effects of various interventions on levels of acridine orange (AO)-labeled and phosphatidylserine (PS)-exposed in CD4 lymphocytes. (**A**-**C**) **AO**-labeled; (**D**-**F**) **PS**-exposed; **HIT**, high-intensity interval training group (**A**, **D**); **MCT**, moderate continuous training group (**B**, **E**) ; **CTL**, control group (**C**, **F**)**; Pre**, pre-intervention; **Post**, post-intervention; **Rest**, resting; **HE**, hypoxic (12%O_2_) exercise test**; Basal**, untreated CD4 lymphocytes (**Day 0**); **Vehicle** and **Rap**, CD4 lymphocytes treated in the absence and presence of rapamycin (500nM) for 24 h (**Day 1**), respectively. *P<0.05, **Rest** vs. **HE**; +P<0.05, **Pre** vs. **Post**; ‡P<0.05, **HIT** or **MCT** vs. **CTL**. Values were mean±SE. n=10 in each group.

**Figure 8:**
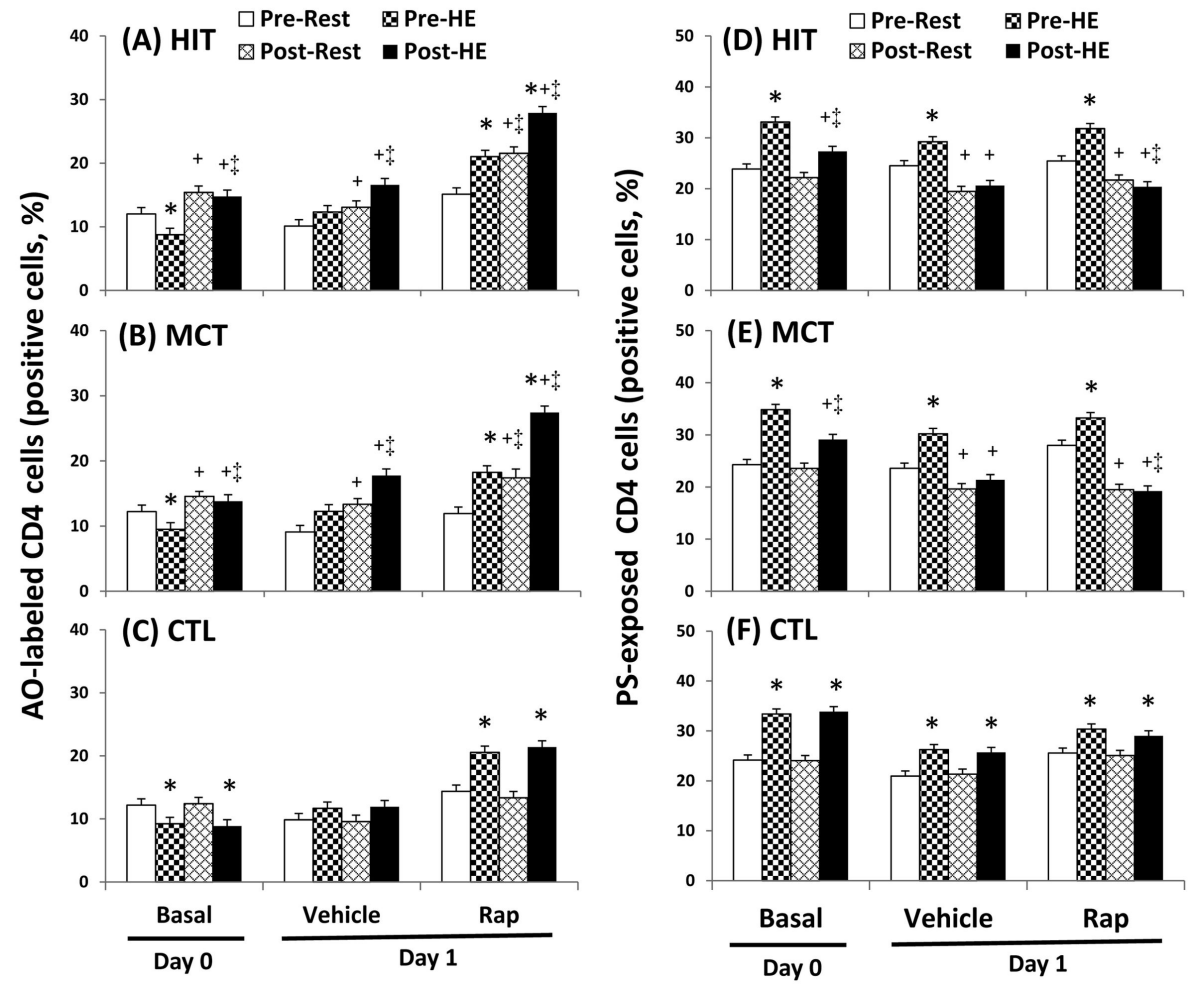
Effects of various interventions on levels of active caspase 9 and caspase 3 in CD4 lymphocytes. (**A**-**C**) caspase 9; (**D**-**F**) caspase 3; **HIT**, high-intensity interval training group (**A**, **D**); **MCT**, moderate continuous training group (**B**, **E**) ; **CTL**, control group (**C**, **F**)**; Pre**, pre-intervention; **Post**, post-intervention; **Rest**, resting; **HE**, hypoxic (12%O_2_) exercise test**; Basal**, untreated CD4 lymphocytes (**Day 0**); **Vehicle** and **Rap**, CD4 lymphocytes treated in the absence and presence of rapamycin (500nM) for 24 h (**Day 1**), respectively. *P<0.05, **Rest** vs. **HE**; +P<0.05, **Pre** vs. **Post**; ‡P<0.05, **HIT** or **MCT** vs. **CTL**. Values were mean±SE. n=10 in each group.

The flow cytometric analysis of HIT (Figures 2A and 3A) and MCT (Figures 2B and 3B) effects on main biomarkers of apoptosis and autophagy [i.e., LAMP-2, active caspase -3, and annexin V staining (PS exposure)] in untreated and rapamycin-treated CD4 lymphocytes are show in Figures 2 and 3, respectively. A bout of 12%O_2_ exercise displayed a decrease in initial autophagy of CD4 lymphocytes, indicated by an increase in Bcl-2 phosphorylation (Figures 4D-4F, P<0.05), down-regulated Atg-1 (Figures 5A-5C, P<0.05) and beclin-1 expressions (Figures 5D-5F, P<0.05). Moreover, HE decreased the levels of Atg-12 (Figures 6A-6C, P<0.05) and LC3-II (Figures 6D-6F, P<0.05) in CD4 lymphocytes, which reflected the diminishing elongation of phagophore membrane and subsequent formation of autophagosome. Additionally, HE reduced LAMP-2 level (Figures 6G-6I, P<0.05) and AO staining (Figures 7A-7C, P<0.05) in CD4 lymphocytes, which were associated with inhibited formation of autophagolysosome that degrades its content through hydrolysis. Conversely, this HE test also enhanced mitochondrial-mediated apoptosis by activating initial caspase-9 (Figures 8A-8C, P<0.05) and execution caspase-3 (Figures 8D-8F, P<0.05), which responses were accompanied by an increase in the PS exposure (Figures 7D-7F, P<0.05) of CD4 lymphocytes. However, increased autophagy-related proteins and PS exposure with unchanging active caspase-9 and -3 contents by HE were observed following the treatment of rapamycin to CD4 lymphocytes for 24 hours (Figures 4, 5, 6, 7, 8).

Five weeks of HIT and MCT reduced the extents of the HE-declined initiation [Atg-1 (Figures 5A and 5B, P<0.05) and Beclin-1 (Figures 5D and 5E, P<0.05)], elongation [Agt-12 (Figures 6A and 6B, P<0.05)], maturation [LC3-II (Figures 6D and 6E, P<0.05)], and fusion [LAMP-2 (Figures 6G and 6H, P<0.05)] of autophagy and potentiated initiation [active caspase 9 (Figures 8A and 8B, P<0.05)] and execution [active caspase 3 (Figures 8D and 8E, P<0.05)] of apoptosis, which were accompanied by a decreases in mTOR (Figures 4A and 4B, P<0.05) and phospho-BcL-2 (Figures 4D and 4E, P<0.05) of CD4 lymphocytes. Furthermore, the two training regimens simultaneously enhanced AO staining (Figures 7A and 7B, P<0.05) and depressed PS exposure (Figures 7D and 7E, P<0.05) in CD4 lymphocytes during HE. Additionally, HIT rather than MCT increased the expressions of Agt-12 (Figure 6A, P<0.05), LC3-II (Figure 6D, P<0.05), and LAMP-2 (Figure 6G, P<0.05) in rapamycin-treated CD4 lymphocyte at rest status. However, no significant changes in autophagy and apoptosis of CD4 lymphocytes treated with or without rapamycin were observed following CTL for 5 weeks (Figures 4, 5, 6, 7, 8).

### Cytokine and peroxide levels in plasma

**Figure 9:**
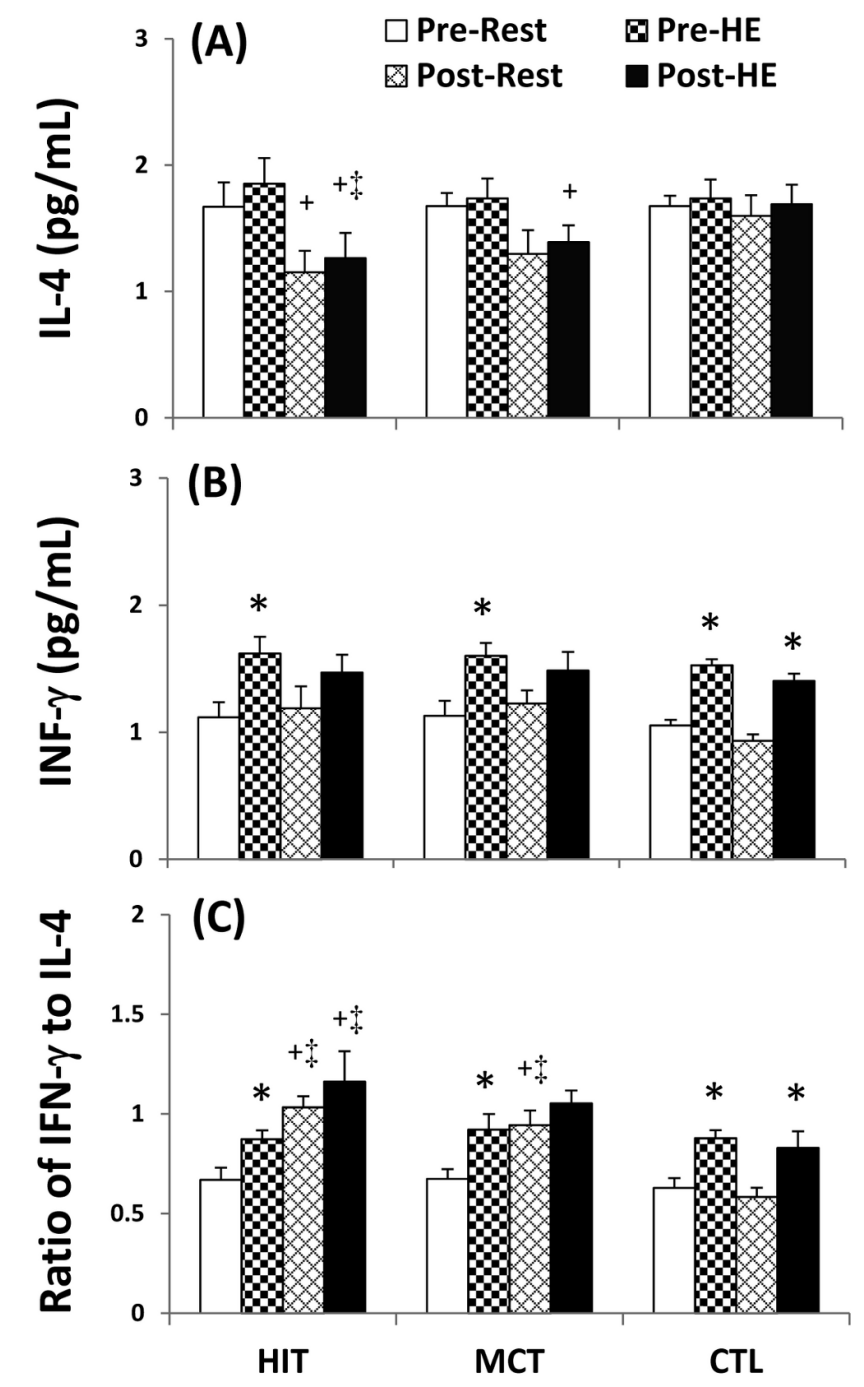
Effects of various interventions on levels of interleukin-4 (IL-4) and interferon-γ (INF-γ) and ratio of IL-4 to INF-γ in plasma. (**A**) IL-4; (**B**) INF-γ; (**C**) ratio of IL-4 to INF-γ; **HIT**, high-intensity interval training group; **MCT**, moderate continuous training group; **CTL**, control group; **Pre**, pre-intervention; **Post**, post-intervention; **Rest**, resting; **HE**, hypoxic (12%O_2_) exercise test. *P<0.05, **Rest** vs. **HE**; +P<0.05, **Pre** vs. **Post**. Values were mean±SE. n=10 in each.

**Figure 10:**
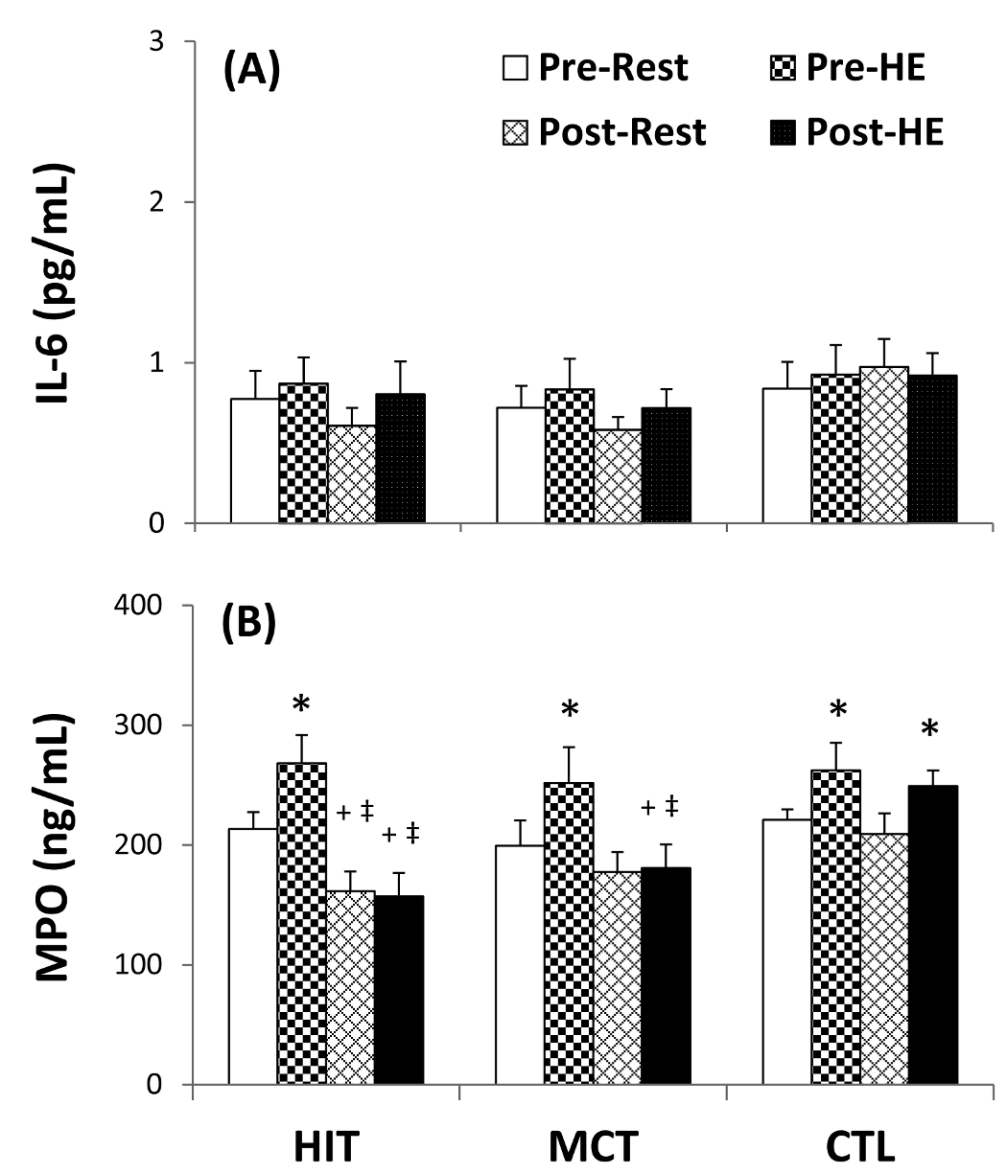
Effects of various interventions on levels of interleukin-6 (IL-6) and myeloperoxidase (MPO) in plasma. (**A**) IL-6; (**B**) MPO; **HIT**, high-intensity interval training group; **MCT**, moderate continuous training group; **CTL**, control group; **Pre**, pre-intervention; **Post**, post-intervention; **Rest**, resting; **HE**, hypoxic (12%O_2_) exercise test. *P<0.05, **Rest** vs. **HE**; +P<0.05, **Pre** vs. **Post**. Values were mean±SE. n=10 in each group.

 At the beginning of the study, acute 12%O_2_ exercise increased IFN-γ (Figure 9B, P<0.05) and unchanged IL-4 levels (Figure 9A, P<0.05), as well as, elevated the ratio of IFN-γ to IL-4, (Figure 9C, P<0.05) in plasma. Five weeks of HIT intervention both increases resting and HE-induced IFN-γ/IL-4 ratio (Figure 9C, P<0.05, d=0.8) by decreasing IL-4 (Figure 9A, P<0.05, d=0.8) rather than changing IFN-γ (Figure 9B) level in plasma. However, same pattern is not observed in the case of MCT intervention whereby only HE-induced IFN-γ/IL-4 ratio increment is detected (Figure 9C, P<0.05). Despite no change in plasma IL-6 level (Figure 10A), the HE significantly increased plasma MPO level (Figure 10B, P<0.05) before various interventions. Nonetheless, the HIT regimen simultaneously decreased resting and HE-induced MPO levels (Figure 10B, P<0.05, d=0.5), whereas the MCT regimen only decreased HE-induced MPO level (Figure 10B, P<0.05). Additionally, there were no significant changes in resting and HE-induced MPO and cytokine levels following 5 weeks of CTL (Figures 9 and 10).

## Discussion

This investigation clearly demonstrates that HIT is more effective for enhancing aerobic fitness of sedentary males by increasing their pulmonary ventilatory and cardiac hemodynamic responses to exercise than MCT. Notably, this study is first to observe that the two training regimens reduce the extents of declined autophagy and potentiated apoptosis of CD4 lymphocytes by 12%O_2_ exercise, which are accompanied by depressed oxidative stress and Th2 cytokine production in blood. By elucidating the relationship between exercise training mode and CD4 lymphocyte autophagy/apoptosis, this study provides a strategy for developing a suitable training regimen that would improve aerobic fitness and minimize risk of immune death evoked by hypoxic stress.

### Effects of exercise training on aerobic fitness

Compared to MCT, HIT for 5 weeks increased CO by increasing SV and decreasing after-load (reflected by increased SVC) in heart at ventilatory threshold. These results imply that the HIT regimen improves the pumping efficiency of the heart at a period of aerobic exercise. Moreover, HIT revealed a larger improvement of V_E_, CO, and VO_2_ at peak performance than MCT, which may represent the superior effects of HIT on the capacity for O_2_ transport and utilization during exercise. Recently, the authors’ study using patients with heart failure revealed that the HIT regimen simultaneously enhanced cerebral/muscular hemodynamics and suppressed oxidative stress/inflammation associated with cardiac dysfunction [19,20]. A clinical investigation further demonstrated that HIT improved mitochondrial biogenesis, as reflected by increased peroxisome proliferative activated receptor-γ coactivator-1α level, in skeletal muscle [25]. These results, together with those presented here, clearly demonstrate that the superior hemodynamic and metabolic adaptations induced by HIT may significantly contribute to improved exercise performance in healthy individuals and patients with cardiovascular diseases.

### CD4 lymphocyte autophagy and apoptosis modulated by HE

This investigation showed that a bout of 12%O_2_ exercise increased phosphor-Bcl-2 and decreased beclin-1 levels, which may reflect to the dissociation of Bcl-2 and beclin-1, subsequently down-regulating Atg1 to depress the initiation of autophagy in CD4 lymphocytes [26]. This HE also decreased the expressions of ubiquitin-like proteins, Atg-12 and LC3-II, and accordingly may diminish the elongation of phagophore membrane, leading to limiting the formation of autophagosome [9]. Additionally, lowered LAMP-2 caused by HE may attenuate the fusion of mature autophagosome with cytosolic lysosomes, eventually inhibiting the development of autophagolysosome [9]. Autophagolysosome degrades its content through hydrolysis and can be detected with AO [9]. Reduced AO staining in CD4 lymphocyte following HE represents a phenomenon as the reduced formation of autophagolysosome by HE. Summarily, HE may substantially suppress systemic autophagy in CD4 lymphocyte throughout the cell initiation, elongation, maturation, and fusion phases. 

Conversely, HE also enhanced mitochondrial-mediated apoptosis by activating initial caspase-9 and subsequently executive caspase-3, which responses were accompanied by an increase in the PS exposure of CD4 lymphocytes. Our early work indicated that 12%O_2_ exercise accelerated senescence and enhanced apoptosis of lymphocytes by consuming cellular anti-oxidative capacity an elevated plasma peroxide level [23]. This study indicated that HE significantly increased plasma MPO concentration. Elevated MPO in plasma can react with the polyunsaturated fatty acids of lipid membranes, resulting in peroxidation of lipid [27]. The present results were consistent with these previous findings [23]. However, HE unchanged plasma IL-6 level in the present study, which is consistent with our previous finding [28]. Actually, the time point of blood collection in this study was immediately after HE, and the IL-6 belongs to the late stage cytokine, and this explains why it is not possible to detect the presence of IL-6 instantly [28]. Furthermore, following the treatment of rapamycin to CD4 lymphocytes for 24 hours, HE only increased the cell autophagy-related protein expressions but did not change initial/executive active caspases contents. These results suggest that the pathway of HE-induced programed death of CD4 lymphocyte transforms from apoptosis to autophagy while inhibiting the cellular mTOR activity [29].

### CD4 lymphocyte autophagy and apoptosis modulated by exercise training

This investigation demonstrated that five weeks of HIT and MCT reduced the extents of HE-declined autophagy and potentiated apoptosis, which responses were accompanied by decreased mTOR and phospho-BcL-2 levels in CD4 lymphocytes. Lowered mTOR expression triggers autophagy to protect cells from oxidative stress [13], whereas eliminated Bcl-2 phosphorylation leads to depressing “initiator” caspase-9 activation and ultimately lowering intrinsic apoptosis [14]. Furthermore, the two exercise regimens significantly lowered plasma MPO and IL-4 levels and elevated the IL-4/IFN-γ ratio during HE. Activated autophagy prevents the cells from undergoing apoptosis by sequestrating damaged mitochondria, thereby diminishing the formation of apoptosomes by decreasing cytochrome c release [29]. Th1 cytokine IFN-γ induces autophagy in an immunity-related GTPase Irgm1-dependent manner [12], whereas Th2 cytokine IL-4 are antagonists of autophagy via activation of the Akt-mTOR cascade [11]. Moreover, IL-4 may offset the effect of IFN-γ to inducing autophagy to anti-microbial functions and cell survival, thereby deteriorating homeostasis of adaptive immune system [11]. Recently, the authors’ study showed that exercise training reduced senescent T-lymphocyte subsets with increasing IFN-γ level and the ratio of IFN-γ to IL-4 in blood, which responses were accompanied by reduced peroxide and pro-inflammatory cytokine productions [21]. However, plasma IL-6 concentration did not change after 5-week HIT or MCT, which may be due to that the training volume is insufficient to influence IL-6 production [21]. Accordingly, we speculate that both HIT and MCT regimens increase autophagy and decreased apoptosis in CD4 lymphocyte by lowering Th2 cytokine production during HE, which may be associated with attenuated the elevation of oxidative stress caused by HE.

At rest status, HIT, but not MCT, exhibited a decrease in plasma MPO level along with increasing expressions of Agt-12, LC3-II, and LAMP-2 in rapamycin-treated CD4 lymphocyte. These results imply that HIT has the superior effects on lowering peroxide production and facilitating autophagy of CD4 lymphocyte than MCT. Successive exercise phases at high intensity and interspersed with moderate-intensity exercise periods, as a form of intermittent ischemic preconditioning, may optimally have a beneficial effect on protecting individuals against oxidative damage [30]. Moreover, exercise or ischemic preconditioning can benefit tissue ischemia-reperfusion injury by preserving the mitochondrial redox state, which protects tissue against reperfusion injury caused by leukocyte-derived oxidants [18,31]. Moreover, maturation and fusion of autophagic pathway mediated by Atg-12, LC3-II, and LAMP-2 with lysosome are critical steps in autophagic degradation and play an important role in response to ischemic reperfusion stress [32]. A recent study showed that upregulated autophagy by rapamycin protected cardiomyocytes from oxidative stress-induced toxicity [13]. By sensitizing mTOR-related lymphocyte autophagy, HIT might be more efficient than MCT in resistance to immune dysfunction evoked by oxidative stress.

### Conclusions

**Figure 11:**
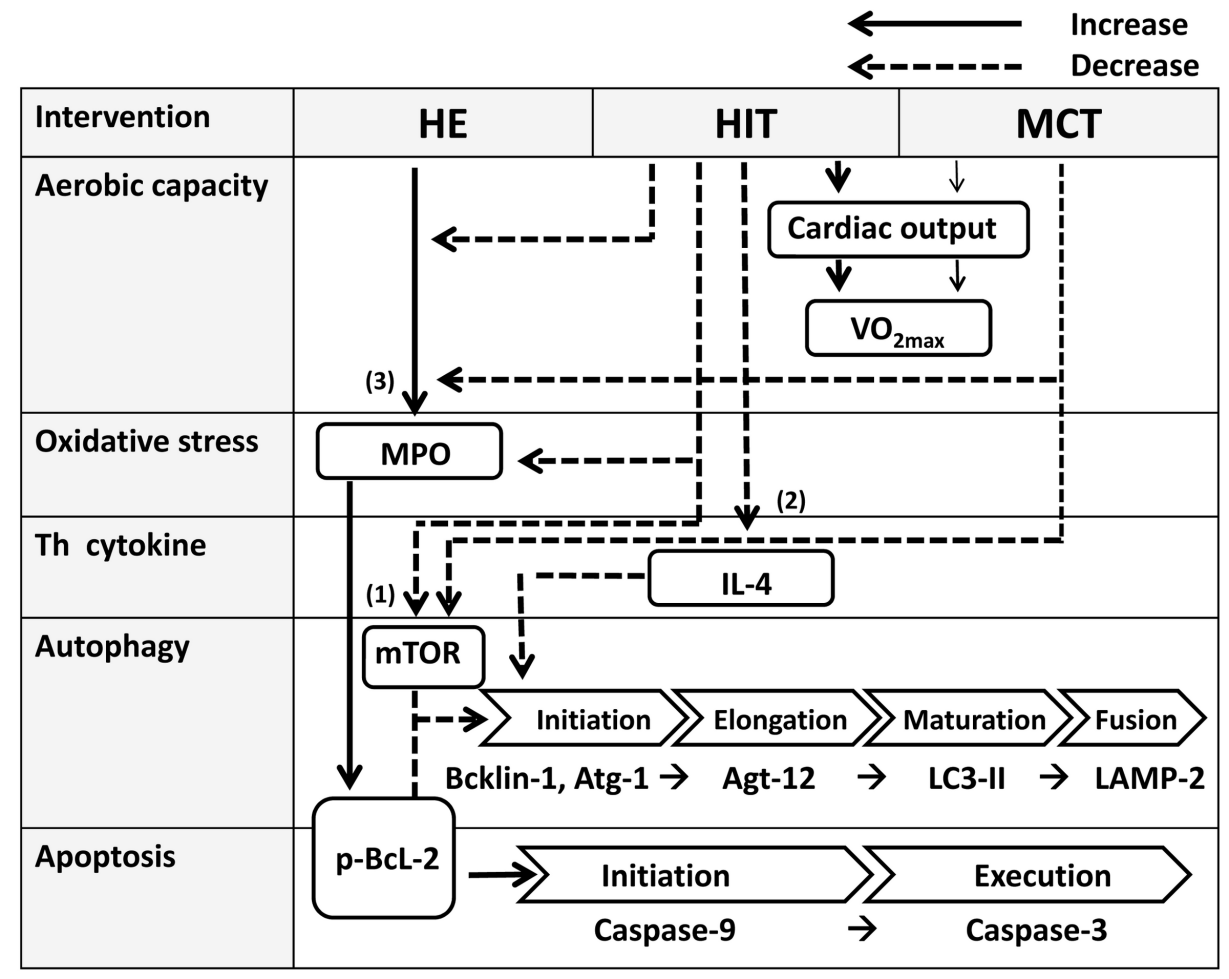
Possible mechanisms of improved aerobic capacity and modulated CD4 lymphocyte autophagic and apoptotic responses to hypoxic stress by various exercise regimens. High-intensity interval training (HIT) is more effective for enhancing aerobic capacity (VO_2max_) by increasing cardiac output response to exercise, compared to moderate continuous training (MCT) does. A bout of 12%O_2_ exercise (HE) suppresses the initiation, elongation, maturation, and fusion of autophagy by decreased beclin-1, Atg-1, LC3-II, Atg-12, and LAMP-2 expressions, and simultaneously enhances the initiation and execution of apoptosis by increased phospho-Bcl-2 and active caspase-9/-3 levels in CD4 lymphocytes. However, five weeks of HIT and MCT attenuate the extents of declined autophagy and potentiated apoptosis in CD4 lymphocyte caused by HE, possibly by (1) down-regulating mTOR expression, (2) lowering Th2 cytokine (indicated by a decrease in interleukin-4, IL-4) production, and (3) depressing elevation of oxidative stress by HE. Besides, HIT, but not MCT, further depresses resting myeloperoxidase (MPO) and IL-4 levels in plasma.

In this investigation, five weeks of HIT exhibits higher enhancements of ventilatory and hemodynamic efficiencies during exercise than the MCT regimen does (Figure 11). Acute HE significantly depresses autophagy by down-regulated beclin-1, Atg-1, LC3-II, Atg-12, LAMP-2, and AO expressions and simultaneously promotes apoptosis by increased phspho-Bcl-2, activated caspase-9 and -3 levels and PS exposure in CD4 lymphocytes. However, both HIT and MCT regimens effectively attenuate the HE-induced autographic depression and apoptotic activation of CD4 lymphocyte, which are associated with lowered blood peroxide and Th2 cytokine productions. Furthermore, HIT is superior to MCT for decreasing resting oxidative stress and elevating ratio of IFN-γ/IL4, which may facilitate autophagosome-lysosome formation (Figure 11). Hence, HIT is a more effective modality for improving aerobic capacity and alleviating CD4 lymphocyte death provoked by hypoxic stress than MCT. These experimental findings facilitate the identification of effective exercise training regimens to increase aerobic capacity and minimize immune death under conditions of hypoxia.

### Study limitation

Our small size in each group (n=10) is a major limitation of this study. However, the aerobic capacity, autophagy, and apoptosis-related results obtained from this investigation have high values of statistical power from 0.907 to 1.000. Additionally, the subjects used tended to be young and healthy, and thus further clinical evidence was required to extrapolate the present results to patients with abnormal or diseased immune systems. This investigation also lacked to measure levels of BAX and BAD in CD4 lymphocytes, which were limited to elucidate the dual roles of phosphorylated Bcl-2 in the cell autophagy and apoptosis. Hence, programmed lymphocyte death caused by Bcl-2-BAX and Bcl-2-BAD interactions requires further study.
